# Cardiometabolic Risk Factors and Incident Cardiovascular Disease Events in Women vs Men With Type 1 Diabetes

**DOI:** 10.1001/jamanetworkopen.2022.30710

**Published:** 2022-09-08

**Authors:** Barbara H. Braffett, Ionut Bebu, Laure El ghormli, Catherine C. Cowie, William I. Sivitz, Rodica Pop-Busui, Mary E. Larkin, Rose A. Gubitosi-Klug, David M. Nathan, John M. Lachin, Samuel Dagogo-Jack

**Affiliations:** 1 The Biostatistics Center, George Washington University, Rockville, Maryland; 2 National Institute of Diabetes and Digestive and Kidney Diseases, Bethesda, Maryland; 3 Division of Endocrinology and Metabolism, University of Iowa, Iowa City; 4 Division of Metabolism, Endocrinology and Diabetes, University of Michigan, Ann Arbor; 5 Massachusetts General Hospital Diabetes Center, Harvard Medical School, Boston; 6 Division of Pediatric Endocrinology, Diabetes and Metabolism, Case-Western Reserve University School of Medicine, Rainbow Babies and Children’s Hospital, Cleveland, Ohio; 7 Division of Endocrinology, Diabetes and Metabolism, University of Tennessee Health Science Center, Memphis

## Abstract

**Importance:**

The lower risk of cardiovascular disease (CVD) among women compared with men in the general population may be diminished among those with diabetes.

**Objective:**

To evaluate cardiometabolic risk factors and their management in association with CVD events in women vs men with type 1 diabetes enrolled in the Diabetes Control and Complications Trial/Epidemiology of Diabetes Interventions and Complications (DCCT/EDIC) study.

**Design, Setting, and Participants:**

This cohort study used data obtained during the combined DCCT (randomized clinical trial, conducted 1983-1993) and EDIC (observational study, conducted 1994 to present) studies through April 30, 2018 (mean [SD] follow-up, 28.8 [5.8] years), at 27 clinical centers in the US and Canada. Data analyses were performed between July 2021 and April 2022.

**Exposure:**

During the DCCT phase, patients were randomized to intensive vs conventional diabetes therapy.

**Main Outcomes and Measures:**

Cardiometabolic risk factors and CVD events were assessed via detailed medical history and focused physical examinations. Blood and urine samples were assayed centrally. CVD events were adjudicated by a review committee. Linear mixed models and Cox proportional hazards models evaluated sex differences in cardiometabolic risk factors and CVD risk over follow-up.

**Results:**

A total of 1441 participants with type 1 diabetes (mean [SD] age at DCCT baseline, 26.8 [7.1] years; 761 [52.8%] men; 1390 [96.5%] non-Hispanic White) were included. Over the duration of the study, compared with men, women had significantly lower body mass index (BMI, calculated as weight in kilograms divided by height in meters squared; β = −0.43 [SE, 0.16]; *P* = .006), waist circumference (β = −10.56 cm [SE, 0.52 cm]; *P* < .001), blood pressure (systolic: β = −5.77 mm Hg [SE, 0.35 mm Hg]; *P* < .001; diastolic: β = −3.23 mm Hg [SE, 0.26 mm Hg]; *P* < .001), and triglyceride levels (β = −10.10 mg/dL [SE, 1.98 mg/dL]; *P* < .001); higher HDL cholesterol levels (β = 9.36 mg/dL [SE, 0.57 mg/dL]; *P* < .001); and similar LDL cholesterol levels (β = −0.76 mg/dL [SE, 1.22 mg/dL]; *P* = .53). Women, compared with men, achieved recommended targets more frequently for blood pressure (ie, <130/80 mm Hg: 90.0% vs 77.4%; *P* < .001) and triglycerides (ie, <150 mg/dL: 97.3% vs 90.5%; *P* < .001). However, sex-specific HDL cholesterol targets (ie, ≥50 mg/dL for women, ≥40 mg/dL for men) were achieved less often (74.3% vs 86.6%; *P* < .001) and cardioprotective medications were used less frequently in women than men (ie, angiotensin-converting enzyme inhibitor or angiotensin II receptor blocker: 29.6% [95% CI, 25.7%-33.9%] vs 40.0% [95% CI, 36.1%-44.0%]; *P* = .001; lipid-lowering medication: 25.3% [95% CI, 22.1%-28.7%] vs 39.6% [95% CI, 36.1%-43.2%]; *P* < .001). Women also had significantly higher pulse rates (mean [SD], 75.2 [6.8] beats per minute vs 71.8 [6.9] beats per minute; *P* < .001) and hemoglobin A_1c_ levels (mean [SD], 8.3% [1.0%] vs 8.1% [1.0%]; *P* = .01) and achieved targets for tighter glycemic control less often than men (ie, hemoglobin A_1c_ <7%: 11.2% [95% CI, 9.3%-13.3%] vs 14.0% [95% CI, 12.0%-16.3%]; *P* = .03).

**Conclusions and Relevance:**

These findings suggest that despite a more favorable cardiometabolic risk factor profile, women with type 1 diabetes did not have a significantly lower CVD event burden than men, suggesting a greater clinical impact of cardiometabolic risk factors in women vs men with diabetes. These findings call for conscientious optimization of the control of CVD risk factors in women with type 1 diabetes.

## Key Points

**Question:**

Are disparities in cardiometabolic risk factors, use of cardioprotective therapies, and achievement of recommended target levels associated with differences in the risk of cardiovascular disease in women vs men with type 1 diabetes?

**Findings:**

In this cohort study of 1441 participants (680 women, 761 men) with type 1 diabetes, women had a more favorable cardiometabolic risk factor profile, received less aggressive cardioprotective medications, and did not demonstrate a significantly lower cardiovascular event burden compared with men.

**Meaning:**

These findings suggest that sex differences in the conventional cardiometabolic risk factor stratification may be associated with diabetes, arguing for recalibration of clinical care guidelines and therapeutic recommendations by sex.

## Teaser

This cohort study assesses sex differences in cardiometabolic risk factors and their management in association with cardiovascular disease (CVD) events in men and women with type 1 diabetes.

## Introduction

In the general population, women have a lower absolute risk of cardiovascular disease (CVD) compared with men.^1,2^ However, among individuals with type 1 or type 2 diabetes, the relative risk of CVD is similar or higher in women compared with men.^2,3,4,5,6^ The exact reasons for the reduction in the advantage in CVD risk among women with diabetes are not fully understood. Data from several studies have shown that women with type 2 diabetes are likely to have a more adverse cardiovascular risk profile than men with type 2 diabetes, in particular suboptimal blood pressure (BP) and lipid levels.^7,8,9,10^ An analysis by Larkin et al^11^ of the Diabetes Control and Complications Trial/Epidemiology of Diabetes Interventions and Complications (DCCT/EDIC) type 1 diabetes cohort in 2010 reported that women were less likely than men to receive treatment with angiotensin converting enzyme inhibitors (ACEIs), angiotensin II receptor blockers (ARBs), or statins, even after adjusting for BP and lipid levels. Reported use of statins was lower in women than men, even in the high-risk subset that had low-density lipoprotein (LDL) cholesterol levels greater than 130 mg/dL (to convert cholesterol to millimoles per liter, multiply by 0.0259). Other studies have also reported less aggressive treatment for women regarding management of CVD risk factors among individuals with type 2 diabetes^9,10^ and those without diabetes.^12,13^ Thus, besides the biological association between diabetes and accelerated atherosclerosis, disparities in the management of risk factors may contribute to the reduced protection for CVD in women with diabetes compared with men with diabetes.

We compared cardiometabolic risk factor profiles, cardioprotective medications use, and incident CVD events in men and women with type 1 diabetes with approximately 29 years of follow-up in the DCCT/EDIC study. We evaluated how temporal trends and increased awareness of diabetes-related complications,^14,15^ including CVD, were associated with the sex disparities findings in CVD risk management reported 10 years previously in the DCCT/EDIC cohort.^11^ Furthermore, with extended follow-up, we examined whether disparities in cardiometabolic risk factors, use of cardioprotective therapies, and achievement of recommended target levels were associated with differences in risk of CVD in women vs men.

## Methods

For this cohort study, institutional review boards at all participating centers approved the protocol. Written informed consent was obtained from all participants. This study is reported following the Strengthening the Reporting of Observational Studies in Epidemiology (STROBE) reporting guideline.

The DCCT/EDIC study has been previously described.^16,17^ Briefly, between 1983 and 1989, 1441 participants with type 1 diabetes (age range, 13-39 years) were randomized to intensive or conventional diabetes therapy in the DCCT to evaluate the effects of glycemia on the development and progression of diabetes-related complications. Participants with a history of CVD, hypertension, or hyperlipidemia were not eligible to participate. After a mean (range) of 6.5 (3-9) years of follow-up, intensive therapy was found to markedly reduce the development and progression of complications. Participants were trained and encouraged to adopt intensive therapy and transitioned to their own health care practitioners for ongoing diabetes care. In 1994, 96% of the surviving DCCT cohort enrolled in the EDIC observational study. Retention rates remained steady throughout EDIC, with 92% of the surviving cohort actively followed on an annual basis as of the data lock. These analyses included all 1441 participants (680 women, 761 men) randomized in the DCCT with follow-up data locked as of April 30, 2018 (mean [SD] follow-up 28.8 [5.8] years).

### DCCT/EDIC Evaluations

Demographic data, including race and ethnicity, were collected during an interview-administered survey. At the DCCT baseline assessment (1983-1989), participants were asked to self-report their “predominant race/ethnicity.” The form was created in 1982 to 1983, and the following 5 categories were given: American Indian or Alaskan Native, Asian or Pacific Islander, Hispanic, non-Hispanic Black, and non-Hispanic White. Race and ethnicity data were collected to describe the cohort.

Quarterly DCCT and annual EDIC evaluations included a detailed medical history and physical examination with measurements of height, weight, waist circumference (only during EDIC), sitting BP, and pulse rate.^16,17^ Pulse pressure was defined as the difference between systolic and diastolic BP readings. Hypertension was defined as systolic BP of 130 mm Hg or greater, diastolic BP of 80 mm Hg or greater, or use of antihypertensive medications. Hyperlipidemia was defined as LDL cholesterol of 130 mg/dL or greater or use of lipid-lowering medications.

Laboratory measurements were performed centrally.^18^ Fasting lipid levels and albumin excretion rates (AERs) were measured annually during DCCT and biennially during EDIC. LDL cholesterol was calculated using the Friedewald equation.^19^ AER was measured from 4-hour urine samples through EDIC year 18 and subsequently from spot urine samples, with calculation of albumin-creatinine ratios.^20^ Estimated glomerular filtration rates (eGFRs) were calculated from serum creatinine measured annually. Kidney disease was defined as an eGFR less than 60 mL/min/1.73 m^2^ on at least 2 consecutive visits, microalbuminuria (AER ≥30 mg per 24 hours on ≥2 consecutive visits), or macroalbuminuria (AER ≥300 mg per 24 hours).

Self-reported concurrent medication use was collected during annual EDIC visits using a standardized form with specific questions concerning use of guidelines-directed therapies, particularly ACEIs, ARBs, β-blockers, calcium channel blockers, and lipid-lowering medications, plus a general question concerning any type of antihypertensive medication. At the end of the form, participants could list medications not previously specified throughout. These medications were then categorized using a publicly available reference data set from the US Food and Drug Administration.

Risk factors were characterized by time-weighted mean values, representing the cumulative exposure since baseline, and calculated by weighting values by the time interval since the last measurement. Categorical classifications of risk factors, based on current treatment guidelines, were also evaluated.^21,22^ Missing data for cardiometabolic risk factors were less than 5%.

All CVD events were centrally adjudicated by a committee masked to treatment assignment and hemoglobin A_1c_ (HbA_1c_). The primary CVD outcome (any CVD) was defined as the time to CVD death or time to the first occurrence of nonfatal myocardial infarction (MI), nonfatal stroke, subclinical MI on electrocardiography, angina confirmed by ischemic changes during exercise tolerance testing or by clinically significant obstruction on coronary angiography, revascularization, or congestive heart failure. The secondary CVD outcome, major adverse cardiovascular event (MACE), was defined as the time to CVD death, nonfatal MI, or nonfatal stroke.^5^

### Statistical Analysis

For quantitative risk factors (eg, body mass index [BMI], calculated as weight in kilograms divided by height in meters squared), generalized linear mixed models (GLMMs) evaluated differences between sexes and rates of change separately by sex, conditional on the within-individual variability. For binary risk factors (eg, medication use), generalized estimating equation models evaluated marginal differences in prevalence between sexes. Separate GLMMs for quantitative risk factors evaluated the difference between sexes in the mean slope over time by testing for an interaction between study year and sex. Separate nested models estimated the mean slope of risk factors by sex. The Kaplan-Meier method estimated the cumulative incidence of the first occurrence of any CVD or MACE by sex. Epanechnikov kernel function provided a smoothed Nelson-Aalen estimate of the underlying hazard function.^23^ Cox proportional hazards regression models estimated the association of sex with risk of CVD, after separate adjustments for risk factors. Risk factors were included in the models as time-dependent covariates, reflecting the value up to the time immediately preceding each event or censoring.^23^ Interactions between sex and risk factors were evaluated. All models were adjusted for age at DCCT baseline and treatment group, and models using waist circumference or medication use were limited to the EDIC period.

All analyses were performed using SAS statistical software version 9.4 (SAS Institute). A 2-sided *P* = .05 was considered statistically significant. Data analyses were performed between July 2021 and April 2022.

## Results

### Cardiometabolic Risk Factors and HbA_1c_ in Women vs Men

A total of 1441 participants with type 1 diabetes (mean [SD] age at DCCT baseline, 26.8 [7.1] years; 761 [52.8%] men) were assessed. In the full cohort, 654 women (96.2%) and 736 men (96.7%) were non-Hispanic White; the remainder of sample included individuals who identified their race and ethnicity as American Indian or Alaskan Native, Asian or Pacific Islander, Hispanic, and non-Hispanic Black. At DCCT baseline, men were approximately 1 year older than women (mean [SD] age, 27.3 [6.9] years vs 26.2 [7.3] years; *P* = .004) with an older age at diabetes onset (mean [SD] age, 21.6 [7.7] years vs 20.2 [8.4] years; *P* = .001) (Table 1). At both DCCT baseline and over the duration of the study, women had significantly higher pulse rates (β = 3.41 [SE, 0.35] beats per minute; *P* < .001) and HbA_1c_ levels (β = 0.13% [SE, 0.05%]; *P* = .01) than men; however, all other significant differences in risk factors demonstrated less atherogenic profiles in women than in men (Table 2). These results were similar for women vs men who did not reach a CVD outcome (eTable 1 in the Supplement). However, among those who did reach a CVD outcome, there were no longer significant differences in age, BMI, or HbA_1c_ by sex.

**Table 1.** Characteristics of Women and Men at Diabetes Control and Complications Trial Baseline

Characteristic	Mean (SD)		*P* value
	Women (n = 680)	Men (n = 761)	
Age, y			
At baseline	26.2 (7.3)	27.3 (6.9)	.004
At diabetes onset	20.2 (8.4)	21.6 (7.7)	.001
Randomized to intensive treatment, No. (%)	345 (50.7)	366 (48.1)	.32
Duration of diabetes, y	6.0 (4.3)	5.7 (4.0)	.33
Current cigarette smoker, No. (%)	117 (17.2)	150 (19.7)	.22
BMI	23.2 (2.9)	23.7 (2.7)	<.001
Blood pressure, mm Hg			
Systolic	110.2 (11.1)	117.6 (11.0)	<.001
Diastolic	70.5 (8.6)	74.4 (8.7)	<.001
Pulse pressure	39.7 (9.6)	43.2 (9.5)	<.001
Pulse rate, bpm	71.1 (10.7)	65.5 (10.8)	<.001
Lipids, mg/dL			
Total cholesterol	180.0 (31.7)	173.2 (34.2)	<.001
HDL cholesterol	54.5 (12.5)	47.0 (10.9)	<.001
LDL cholesterol	110.2 (27.7)	109.2 (30.3)	.35
Triglycerides^a^	76.6 (36.4)	85.6 (55.3)	.002
Sustained AER ≥30 mg/24 h, No. (%)	30 (4.4)	38 (5.0)	.60
Glycemic control			
HbA_1c_, %	9.1 (1.7)	8.7 (1.5)	<.001
HbA_1c_, mmol/mol	75.6 (18.0)	72.0 (16.6)	<.001

Abbreviations: AER, albumin excretion rate; BMI, body mass index (calculated as weight in kilograms divided by height in meters squared); HbA_1c_, hemoglobin A_1c_; HDL, high-density lipoprotein; LDL, low-density lipoprotein.

SI conversion factors: To convert cholesterol to millimoles per liter, multiply by 0.0259; HbA_1c_ to proportion of total hemoglobin, multiply by 0.01; and triglycerides to millimoles per liter, multiply by 0.0113.

^a^ *P* values for differences by sex were calculated with the Wilcoxon rank-sum test for continuous variables or the χ^2^ test for categorical variables.

**Table 2.** Mean Differences in Longitudinal Cardiometabolic Risk Factors and HbA_1c_ Between Women and Men During Diabetes Control and Complications Trial/Epidemiology of Diabetes Interventions and Complications

Measure	Mean (SD)		Women vs men comparison^a^		
	Women (n = 680)	Men (n = 761)	β (SE)	*t* value/*z* value^b^	*P* value
BMI	25.1 (3.0)	25.6 (3.0)	−0.43 (0.16)	−2.75	.006
Waist circumference, cm	82.5 (10.0)	93.1 (10.1)	−10.56 (0.52)	−20.43	<.001
Blood pressure, mm Hg					
Systolic	113.1 (6.9)	118.9 (6.9)	−5.77 (0.35)	−16.55	<.001
Diastolic	72.3 (5.1)	75.5 (5.2)	−3.23 (0.26)	−12.41	<.001
Pulse pressure	40.9 (5.1)	43.5 (5.2)	−2.58 (0.26)	−9.94	<.001
Pulse rate, bpm	75.2 (6.8)	71.8 (6.9)	3.41 (0.35)	9.85	<.001
Any hypertension, % (95% CI)^c^	90.5 (88.2-92.4)	95.3 (93.8-96.4)	−0.75 (0.16)	−4.62	<.001
Lipids, mg/dL					
Total cholesterol	186.0 (26.0)	179.4 (26.0)	6.63 (1.37)	4.83	<.001
HDL cholesterol	58.8 (11.1)	49.4 (11.2)	9.36 (0.57)	16.35	<.001
LDL cholesterol	111.4 (23.1)	112.2 (23.2)	−0.76 (1.22)	−0.62	.53
Triglycerides^d^	79.4 (39.4)	89.5 (39.7)	−10.10 (1.98)	−5.11	<.001
Any hyperlipidemia, % (95% CI)^c^	58.9 (55.2-62.5)	57.1 (53.8-60.2)	0.07 (0.11)	0.70	.49
Medications, % (95% CI)					
ACEIs or ARBs	29.6 (25.7-33.9)	40.0 (36.1-44.0)	−0.46 (0.14)	−3.20	.001
β-blockers	4.4 (3.5-5.6)	5.1 (4.1-6.3)	−0.14 (0.16)	−0.86	.39
Calcium channel blockers	5.2 (4.1-6.6)	5.9 (4.7-7.5)	−0.14 (0.19)	−0.74	.46
Lipid-lowering	25.3 (22.1-28.7)	39.6 (36.1-43.2)	−0.66 (0.12)	−5.75	<.001
Kidney disease, % (95% CI)^c^					
Any sustained eGFR<60 mL/min/1.73 m^2^	2.4 (1.7-3.5)	2.9 (2.1-4.1)	−0.19 (0.20)	−0.93	.35
Any sustained AER ≥30 mg/24 h	16.2 (12.8-20.4)	23.8 (20.3-27.8)	−0.48 (0.20)	−2.40	.02
Any AER ≥300 mg/24 h	6.0 (4.6-7.8)	12.5 (10.5-14.9)	−0.80 (0.17)	−4.71	<.001
Glycemic control					
HbA_1c_, %	8.3 (1.0)	8.1 (1.0)	0.13 (0.05)	2.46	.01
HbA_1c_, mmol/mol	66.7 (10.8)	65.3 (10.9)	1.41 (0.57)	2.46	.01

Abbreviations: ACEI, angiotensin-converting enzyme inhibitor; AER, albumin excretion rate; ARB, angiotensin II receptor blocker; BMI, body mass index (calculated as weight in kilograms divided by height in meters squared); eGFR, estimated glomerular filtration rate; HbA_1c_, hemoglobin A_1c_; HDL, high-density lipoprotein; LDL, low-density lipoprotein.

SI conversion factors: To convert cholesterol to millimoles per liter, multiply by 0.0259; HbA_1c_ to proportion of total hemoglobin, multiply by 0.01; and triglycerides to millimoles per liter, multiply by 0.0113.

^a^ Separate linear mixed models and generalized estimating equation models assessing the differences between sexes (women vs men) in the mean of each quantitative risk factor or the prevalence of each binary risk factor over repeated time points. Diabetes Control and Complications Trial/Epidemiology of Diabetes Interventions and Complications study year was included in the models as a class effect. Each model was adjusted for Diabetes Control and Complications Trial baseline age and treatment group. β estimates are equal to the mean difference between women and men for quantitative risk factors or the log odds ratio for binary risk factors. The signed *t*-value for continuous factors and *z*-value for binary factors correspond to the magnitude and directionality of the association. SDs were estimated from the SEs of the adjusted β using the equation, SD = SE × SQRT(N).

^b^ *t* Values were calculated from linear mixed models for continuous variables, and *z*-vales were calculated from generalized estimating equations for binary risk factors.

^c^ Any report between Diabetes Control and Complications Trial baseline and each study visit.

^d^ *P* value from model based on the log-transformed value.

Compared with men, women had lower BMI (β = −0.43 [SE, 0.16]; *P* = .006), waist circumference (β = −10.56 [SE, 0.52] cm; *P* < .001), BP (systolic: β = −5.77 [SE, 0.35] mm Hg; *P* < .001; diastolic: β = −3.23 [SE, 0.26] mm Hg; *P* < .001), and triglycerides levels (β = −10.10 [SE, 1.98] mg/dL; *P* < .001) (to convert to millimoles per liter, multiply by 0.0113). Women had higher HDL cholesterol levels (β = 9.36 [SE, 0.57] mg/dL; *P* < .001) and similar LDL cholesterol levels (β = −0.76 [SE, 1.22] mg/dL; *P* = .53) (Table 2). Hypertension and kidney disease occurred less often among women compared with men. The significant sex differences in risk factors persisted after adjustment for the corresponding medication use.

Medication use was reported less often among women compared with men, specifically, use of ACEIs or ARBs (29.6% [95% CI, 25.7%-33.9%] vs 40.0% [95% CI, 36.1%-44.0%]; *P* = .001) and lipid-lowering medications (25.3% [95% CI, 22.1%-28.7%] vs 39.6% [95% CI, 36.1%-43.2%]; *P* < .001) (Table 2). The findings persisted when the analyses were restricted to participants with elevated BPs or elevated LDL cholesterol levels. Treatment with medication was reported less often among women compared with men among the subset of participants with elevated BPs (ACEIs or ARBs: 30.8% vs 39.9%; *P* = .006) or elevated LDL cholesterol levels (lipid-lowering medications: 52.9% vs 67.4%; *P* < .001).

Recommended targets for risk factor control were achieved more frequently among women compared with men, including optimal BMI (ie, <25: 56.4% [95% CI, 53.0%-59.8%] vs 47.6% [95% CI, 44.5%-50.7%]; *P* < .001), BP (ie, <130/80 mm Hg: 90.0% [95% CI, 87.5%-92.0%] vs 77.4% [95% CI, 74.6%-80.0%]; *P* < .001), and triglycerides (ie, <150 mg/dL: 97.3% [95% CI, 95.5%-98.3%] vs 90.5% [95% CI, 88.3%-92.3%]; *P* < .001) (Table 3). Despite higher longitudinal HDL cholesterol levels, recommended sex-specific targets for HDL cholesterol were achieved less often among women compared with men (ie, ≥50 mg/dL for women or ≥40 mg/dL for men: 74.3% [95% CI, 70.8%-77.6%] vs 86.6% [95% CI, 83.4%-89.2%]; *P* < .001). There was a significant difference between sexes in the proportion achieving recommended targets for HbA_1c_ (ie, <7%), which favored men (11.2% [95% CI, 9.3%-13.3%] of women vs 14.0% [95% CI, 9.3%-13.3%] of men; *P* = .03).

**Table 3.** Longitudinal Prevalence of Achieving Target Levels in Women and Men and Associations With Cardiovascular Disease Risk During Diabetes Control and Complications Trial/Epidemiology of Diabetes Interventions and Complications

Measure	% (95% CI)		*P* value^a^	Hazard ratio (95% CI)^b^	
	Among women (n = 680)	Among men (n = 761)		Any CVD	MACE
BMI					
<25	56.4 (53.0-59.8)	47.6 (44.5-50.7)	<.001	0.80 (0.61-1.03)	1.14 (0.81-1.62)
<30	92.2 (90.0-94.0)	93.2 (91.2-94.7)	.53	0.74 (0.54-1.01)	0.95 (0.61-1.48)
Waist <88 cm (women) or <102 cm (men)	79.1 (75.0-82.6)	81.3 (77.4-84.7)	.49	0.65 (0.49-0.86)	0.69 (0.47-1.01)
Systolic/diastolic BP <130/80 mm Hg	90.0 (87.5-92.0)	77.4 (74.6-80.0)	<.001	0.63 (0.48-0.83)	0.80 (0.54-1.19)
Lipids					
Cholesterol					
Total <200 mg/dL	73.9 (70.7-76.8)	79.1 (76.4-81.5)	.01	0.53 (0.41-0.69)	0.51 (0.36-0.73)
HDL ≥50 mg/dL (women) or ≥40 mg/dL (men)	74.3 (70.8-77.6)	86.6 (83.4-89.2)	<.001	0.66 (0.49-0.88)	0.52 (0.35-0.75)
LDL <100 mg/dL	31.5 (28.5-34.6)	32.2 (29.3-35.2)	.74	0.70 (0.53-0.94)	0.74 (0.50-1.10)
Triglycerides <150 mg/dL	97.3 (95.5-98.3)	90.5 (88.3-92.3)	<.001	0.43 (0.30-0.62)	0.34 (0.22-0.53)
HbA_1c_ <7%	11.2 (9.3-13.3)	14.0 (12.0-16.3)	.03	0.55 (0.36-0.86)	0.63 (0.35-1.13)

Abbreviations: BMI, body mass index (calculated as weight in kilograms divided by height in meters squared); BP, blood pressure; CVD, cardiovascular disease; HbA_1c_, hemoglobin A_1c_; HDL, high-density lipoprotein; LDL, low-density lipoprotein; MACE, major adverse cardiovascular event.

SI conversion factors: To convert cholesterol to millimoles per liter, multiply by 0.0259; HbA_1c_ to proportion of total hemoglobin, multiply by 0.01; and triglycerides to millimoles per liter, multiply by 0.0113.

^a^ Separate generalized estimating equation models assessing the differences between sexes (women vs men) in the prevalence of each binary risk factor over repeated time points. Diabetes Control and Complications Trial/Epidemiology of Diabetes Interventions and Complications study year was included in the models as a class effect. Each model was adjusted for Diabetes Control and Complications Trial baseline age and treatment group.

^b^ Separate Cox proportional hazard regression models adjusted for Diabetes Control and Complications Trial baseline age and treatment group. An interaction between sex and target level was evaluated in each model.

Among the subset of participants who did not achieve recommended targets for control of BP or triglycerides, there were no significant differences between sexes in the proportion reporting treatment with ACEIs or ARBs or lipid-lowering medications. However, treatment with lipid-lowering medications was reported less often among women compared with men among the subset of participants who did not achieve treatment targets for LDL cholesterol (30.9% vs 44.6%; *P* < .001).

Systolic BP and pulse pressure increased over the duration of the study in both women and men, yet the mean slope (rate of change per year) was significantly higher in women compared with men (eTable 2 and eFigure in the Supplement). Pulse rate decreased in women and increased in men, while HDL cholesterol increased in both women and men, with a higher mean slope observed in women compared with men. There were no statistically significant sex differences in HbA_1c_ slopes.

### CVD Outcomes in Women vs Men

During DCCT/EDIC, 113 women (16.6%) and 150 men (19.7%) experienced at least 1 CVD event, including 57 women (8.4%) and 82 men (10.8%) who experienced at least 1 MACE. There was no significant difference between sexes in risk of any CVD (unadjusted HR, 0.81; 95% CI, 0.64-1.04) or MACE (unadjusted HR, 0.74; 95% CI, 0.53-1.03) (Figure, A and C). The hazard rate per year of any CVD increased at a similar rate in both women and men until approximately the 18th year of follow-up (log-rank *P* = .67), when risk of CVD began to increase in men more rapidly than in women (log-rank *P* = .03) (Figure, B and D).

**Figure.**
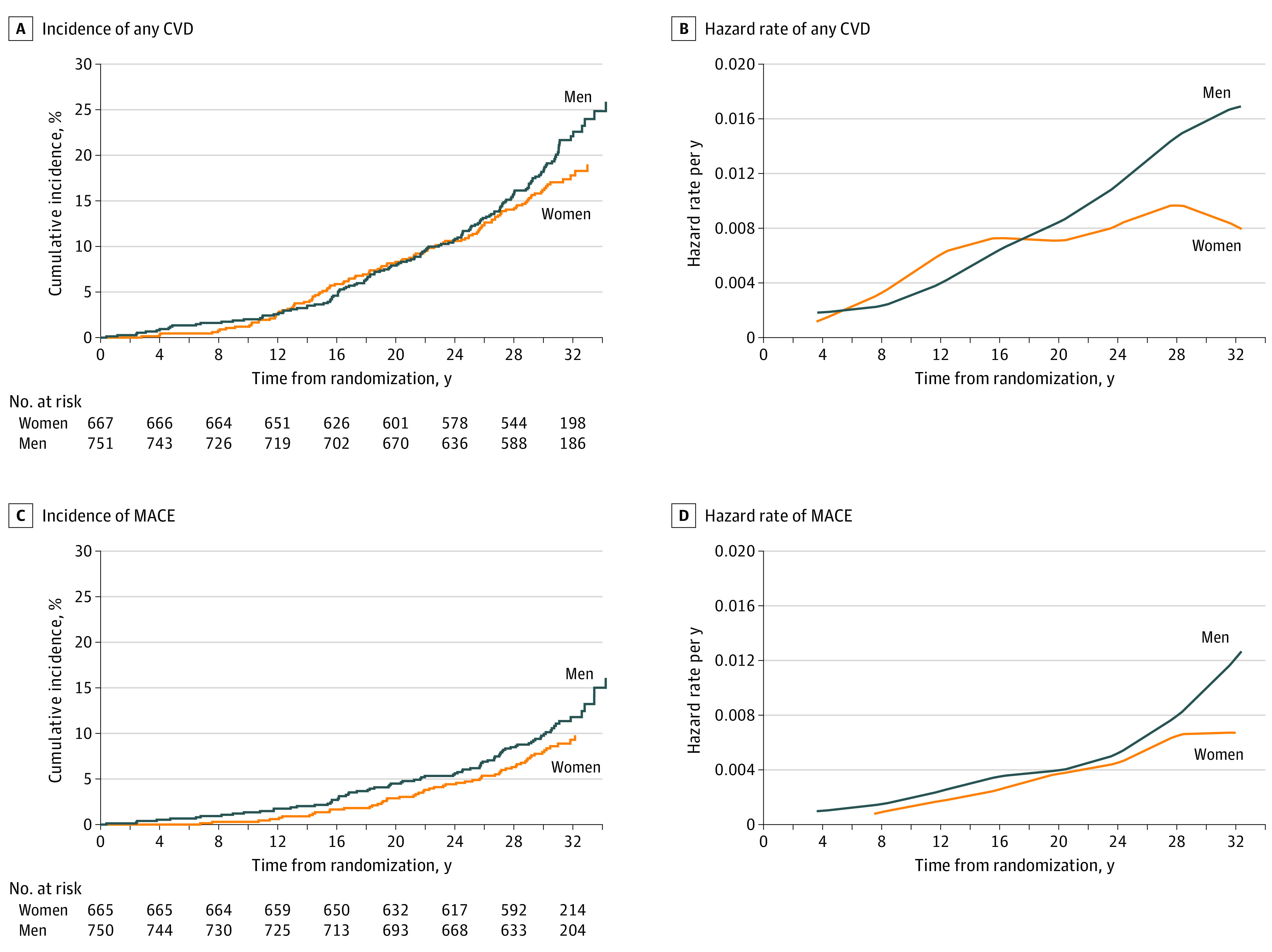
Cumulative Incidence and Smoothed Estimate of the Incidence Rate of the First Occurrence of Any Cardiovascular Disease (CVD) Event and Major Adverse Cardiovascular Event (MACE) for Women vs Men The unadjusted log-rank *P* values for women vs men were *P* = .10 for any CVD and *P* = .08 for MACE.

In models adjusted separately for risk factors shown in Table 2, there remained no statistically significant difference between women and men in the risk of any CVD or MACE in the presence of each factor except for pulse rate (eTable 3 in the Supplement). Models adjusted for pulse rate yielded a significantly decreased risk of any CVD (HR, 0.75; 95% CI, 0.59-0.97) and of MACE (HR, 0.66; 95% CI, 0.47-0.94) among women compared with men, which persisted after further adjustment for HbA_1c_. There were no other significant differences in the presence of each risk factor further adjusted for HbA_1c_.

### Cardiometabolic Risk Factor Targets and CVD in Women vs Men

Achieving treatment guidelines for waist circumference, BP, lipids, and HbA_1c_ was associated with 30% to 57% reduction in the risk of any CVD, while achieving lipid targets was associated with a 26% to 66% risk reduction in MACE (Table 3). Higher BP, LDL cholesterol, and HbA_1c_ were associated with increased risk of any CVD and MACE (Table 4).

**Table 4.** Associations Between Risk Factor Levels or Targets and CVD Risk During Diabetes Control and Complications Trial/Epidemiology of Diabetes Interventions and Complications in Women and Men

Factor	% (95% CI)		Hazard ratio (95% CI)^a^			
	Women	Men	Any CVD		MACE	
			Women	Men	Women	Men
Risk factors, mean (SD)						
BP, mm Hg^b^						
Systolic	113.1 (6.9)	118.9 (6.9)	1.36 (1.22-1.52)	1.20 (1.09-1.33)	1.45 (1.24-1.69)	1.11 (0.97-1.27)^c^
Diastolic	72.3 (4.9)	75.5 (5.0)	1.33 (1.10-1.60)	1.24 (1.06-1.46)	1.40 (1.07- 1.83)	1.01 (0.81-1.26)
LDL cholesterol, mean (SD), mg/dL^d^	110.2 (27.7)	109.2 (30.3)	1.18 (1.09-1.27)	1.13 (1.06-1.22)	1.26 (1.12-1.41)	1.06 (0.96-1.17)^b^
HbA_1c_, %^e^	8.3 (1.0)	8.1 (1.0)	1.61 (1.38-1.89)	1.53 (1.31-1.80)	2.19 (1.76-2.72)	1.55 (1.25-1.91)^c^
Risk factor targets						
Systolic/diastolic BP <130/80 mm Hg	90.0 (87.5-92.0)	77.4 (74.6-80.0)	0.58 (0.36-0.94)	0.63 (0.45-0.88)	0.79 (0.38-1.63)	0.80 (0.50-1.27)
LDL cholesterol <100 mg/dL	31.5 (28.5-34.6)	32.2 (29.3-35.2)	0.54 (0.34-0.86)	0.85 (0.58-1.24)	0.40 (0.19-0.81)	1.08 (0.67-1.76)^c^
HbA_1c_ <7%	11.2 (9.3-13.3)	14.0 (12.0-16.3)	0.44 (0.20-0.96)	0.62 (0.36-1.05)	0.42 (0.13-1.35)	0.74 (0.37-1.47)
Risk factor profiles						
≥1 Risk factor elevated	96.9 (95.7-97.8)	96.1 (94.7-97.2)	5.26 (0.73-37.7)	0.88 (0.38-2.00)	NA^f^	0.36 (0.16-0.79)
≥2 Risk factors elevated	69.0 (66.0-71.8)	69.6 (66.7-72.3)	2.17 (1.34-3.52)	2.09 (1.34-3.24)	3.25 (1.47-7.18)	1.54 (0.89-2.67)

Abbreviations: BP, blood pressure; CVD, cardiovascular disease; HbA_1c_, hemoglobin A_1c_; LDL, low-density lipoprotein; MACE, major adverse cardiovascular event; NA, not applicable.

SI conversion factors: To convert cholesterol to millimoles per liter, multiply by 0.0259; HbA_1c_ to proportion of total hemoglobin, multiply by 0.01.

^a^ Separate stratified Cox proportional hazard regression models adjusted for Diabetes Control and Complications Trial baseline age and treatment group. An interaction between sex and risk factor was evaluated in each model.

^b^ Hazard ratios calculated per 5–mm Hg increase.

^c^ Significant interaction between sex and risk factor.

^d^ Hazard ratios calculated per 10-mg/dL increase.

^e^ Hazard ratios calculated per 1–percentage point increase.

^f^ Model did not converge.

## Discussion

This cohort study assessed sex differences in achieving recommended CVD risk management targets and associations with CVD events. We previously described sex disparities in use of cardioprotective medications^11^ and the longitudinal associations between glycemia and traditional CVD risk factors^24^ in the DCCT/EDIC study. The major strengths of this study include the multicenter prospective design of DCCT/EDIC, the extensive longitudinal phenotypic characterization of participants, and the rigorous adjudication of CVD events.

Our findings demonstrated that the prevalence and mean levels of most cardiometabolic risk factors (except for pulse rate and HbA_1c_) were consistent with a less atherogenic profile among women compared with men. Consistent with our 2010 findings,^11^ women were still less likely to receive standard cardioprotective medications, despite emerging evidence since 2010 regarding increased CVD risk in women. Also importantly, we found that achieving treatment targets for BP, lipids, and glucose was associated with significantly decreased risk of CVD in both women and men, a finding that underscores the need for optimal control of comorbid risk factors in all individuals with diabetes.

We previously reported that age and mean HbA_1c_ were associated with risk of CVD and with other conventional risk factors, such as BP, lipids, and lack of ACEI use^5^; however, we found no significant differences between women and men. In this study, with additional follow-up and event accrual, we found that women continued to have statistically similar CVD rates as men. We also observed that the hazard rate per year for any CVD began to increase in men more rapidly than in women after approximately 18 years of follow-up; however, there were no sex differences in risk factor profiles after 18 years. In addition, the dropout rate over the course of the study did not differ by sex.

While the protection for CVD in women vs men in the general population is reduced in the presence of diabetes, several studies have found that the absolute risk of CVD remains similar or lower in women with diabetes compared with men with diabetes.^4,25,26,27^ However, several meta-analyses of prospective cohort studies have shown that the relative risk of CVD mortality and morbidity associated with type 2 diabetes is higher in women than in men,^28,29,30,31^ suggesting that the increase in risk associated with type 2 diabetes is higher in women than in men. The relative and absolute risks in type 1 diabetes are less clear; however, the diminished protection associated with both type 1 and type 2 diabetes may be driven by greater adverse interactions between risk factors and diabetes in the women than in men.^2,3,4,27,28,30,32^

Although women with type 1 diabetes had a more favorable CVD risk factor profile than men with type 1 diabetes in our study, the benefit was narrower (smaller differences) than that observed in the general population. The Framingham Heart Study^33^ found that women, compared with men, had lower systolic BP (118 mm Hg vs 126 mm Hg), diastolic BP (76 mm Hg vs 82 mm Hg), total cholesterol (192 mg/dL vs 202 mg/dL), LDL cholesterol (120 mg/dL vs 135 mg/dL), BMI (23.9 vs 26.5), and triglycerides (77 mg/dL vs 115 mg/dL), and higher HDL cholesterol (57 mg/dL vs 44 mg/dL). All of these sex differences were associated with reduced risk of CVD but were greater than those observed in the more contemporaneous DCCT/EDIC study. Therefore, although women in the DCCT/EDIC cohort presented with a better risk factor profile than men, diabetes may be decreasing the reduced risk of CVD observed among women without diabetes.

Consistent with this notion, type 2 diabetes is associated with a disproportionately higher CVD mortality in women compared with men.^34^ The mechanisms of the female-specific impact of diabetes on CVD risk are not fully understood. Compared with men, women with diabetes have uniquely less favorable CVD risk factors, including endothelial dysfunction, hypercoagulability, and dysfibrinolysis.^2,35,36,37^ Another possible explanation for the lack of protection among women despite better risk factor profiles could be the lower use of medications compared with men. Our analyses restricted to participants with elevated BP or LDL cholesterol levels did confirm lower use of guidelines-directed cardioprotective medications in women vs men.^21^

HDL cholesterol levels are typically higher in women than men; however, a 2011 study^38^ demonstrated that the protective associations of HDL cholesterol levels greater than 50 mg/dL may be markedly attenuated in women vs men with type 1 diabetes. We found that despite higher longitudinal HDL cholesterol levels, women achieved recommended sex-specific targets for HDL cholesterol less often than men. Achieving treatment guidelines for HDL cholesterol was associated with significantly reduced risk of any CVD in men but not in women. We speculate that a policy of more aggressive control of CVD risk factors in women with diabetes, targeting goals that are more stringent than currently recommended, may prove beneficial.

### Limitations

This study has some limitations. The major weakness of the study is the predominantly non-Hispanic White population which, while typical for type 1 diabetes in the US, limits the generalizability to other populations. In addition, measurements of waist circumference and medication use were not available during the DCCT; however, the currently available cardiorenal protective agents, such as ACEIs (approved in 1981), statins (approved in 1987), and ARBs (approved in 2010) were either unavailable or not widely prescribed during the DCCT period. Furthermore, prescription of medications other than insulin was at the discretion of participants’ community physicians, not DCCT/EDIC investigators. Several factors, including the perception of lower CVD risk in women and sex differences in CVD risk estimation calculators, could have influenced the decision to initiate cardioprotective medications. Thus, although we could not control the prescription of medications, our findings probably reflect practice patterns in the community. Additionally, we cannot exclude the possibility that with a larger number of CVD events, the sex difference in risk could become statistically significant.

## Conclusions

The findings of this cohort study suggest that compared with men with type 1 diabetes, women with type 1 diabetes may have a lower burden of cardiometabolic risk factors, receive less aggressive cardioprotective medications (despite current guidelines), and have no advantage associated with sex in CVD outcomes. Our findings argue for a recalibration of CVD risk factor stratification in revised clinical care guidelines and therapeutic recommendations by sex for individuals with type 1 diabetes. The need for conscientious optimization of the control of comorbid risk factors in women with diabetes cannot be overstated.

## Notes

Supplement. **eTable 1.** Characteristics of Women and Men at DCCT Baseline, by Presence or Absence of Any CVD **eTable 2.** Rate of Change of Cardiometabolic Risk Factors and HbA_1c_ During DCCT/EDIC for Women and Men **eTable 3.** Risk of Cardiovascular Disease in Women vs Men After Individual Adjustment for Cardiometabolic Risk Factors **eTable 4.** Associations Between Risk Factor Levels or Targets and Cardiovascular Disease Risk During DCCT/EDIC in Women and Men **eFigure.** Mean Slope During EDIC for Women vs Men **eAppendix.** DCCT/EDIC Research Group
