# Supplementary material for: Cardiometabolic Risk Factors and Incident Cardiovascular Disease Events in Women vs Men With Type 1 Diabetes
Source: JAMA Netw Open. 2022 Sep 8;5(9):e2230710. doi: 10.1001/jamanetworkopen.2022.30710 (PMC9459657; doi:10.1001/jamanetworkopen.2022.30710)
Supplement: Supplement. — eTable 1. Characteristics of Women and Men at DCCT Baseline, by Presence or Absence of Any CVD eTable 2. Rate of Change of Cardiometabolic Risk Factors and HbA1c During DCCT/EDIC for Women and Men eTable 3. Risk of Cardiovascular Disease in Women vs Men After Individual Adjustment for Cardiometabolic Risk Factors eTable 4. Associations Between Risk Factor Levels or Targets and Cardiovascular Disease Risk During DCCT/EDIC in Women and Men eFigure. Mean Slope During EDIC for Women vs Men eAppendix. DCCT/EDIC Research Group [file jamanetwopen-e2230710-s001.pdf]

Supplemental Online Content

Braffett BH, Bebu I, El ghormli L, et al; DCCT/EDIC Research Group. Cardiometabolic risk factors and incident cardiovascular disease events in women vs men with type 1 diabetes. *JAMA Netw Open*. 2022;5(9):e2230710. doi:10.1001/jamanetworkopen.2022.30710

eTable 1. Characteristics of Women and Men at DCCT Baseline, by Presence or Absence of Any CVD

eTable 2. Rate of Change of Cardiometabolic Risk Factors and HbA_{1c} During DCCT/EDIC for Women and Men

eTable 3. Risk of Cardiovascular Disease in Women vs Men After Individual Adjustment for Cardiometabolic Risk Factors

eTable 4. Associations Between Risk Factor Levels or Targets and Cardiovascular Disease Risk During DCCT/EDIC in Women and Men

eFigure. Mean Slope During EDIC for Women vs Men

eAppendix. DCCT/EDIC Research Group

This supplemental material has been provided by the authors to give readers additional information about their work.

© 2022 Braffett BH et al. *JAMA Network Open.*

eTable 1. eTable 1. Characteristics of Women and Men at DCCT Baseline, by Presence or Absence of Any CVD

	Any CVD					
	Yes			No		
	**Women (N=113)**	**Men (N=150)**		**Women (N=567)**	**Men (N=611)**	
**Demographics**	Mean (SD) or %		p-value[a]			p-value[a]
Age, years	29.8 (6.3)	30.0 (6.4)	0.69	25.5 (7.3)	26.6 (6.8)	0.005
Age at diabetes onset, years	22.9 (8.1)	23.7 (8.0)	0.43	19.7 (8.3)	21.1 (7.6)	0.002
Intensive treatment, %	49.6	46.7	0.64	51.0	48.4	0.39
Primary prevention, %	41.6	39.3	0.71	53.1	52.2	0.76
Duration of diabetes, years	6.9 (4.5)	6.3 (4.2)	0.29	5.8 (4.2)	5.6 (4.0)	0.48
Current cigarette smoker, %	30.1	26.7	0.54	14.6	18.0	0.12
**Physical**						
BMI, kg/m^2	23.9 (3.0)	24.4 (2.8)	0.15	23.0 (2.8)	23.5 (2.6)	0.001
**Blood Pressure**						
Systolic, mmHg	112.6 (11.4)	118.7 (10.7)	<0.001	109.7 (10.9)	117.3 (11.1)	<0.001
Diastolic, mmHg	72.2 (7.9)	74.7 (8.9)	0.01	70.1 (8.7)	74.3 (8.6)	<0.001
Pulse pressure, mmHg	40.3 (9.8)	44.0 (9.7)	0.003	39.5 (9.5)	43.0 (9.4)	<0.001
Pulse rate, bpm	73.0 (11.3)	65.9 (10.8)	<0.001	70.7 (10.5)	65.4 (10.9)	<0.001
**Lipids**						
Total cholesterol, mg/dl	186.5 (32.4)	181.5 (35.7)	0.10	178.8 (31.4)	171.1 (33.6)	<0.001
HDL cholesterol, mg/dl	53.4 (12.6)	45.4 (11.1)	<0.001	54.8 (12.5)	47.4 (10.9)	<0.001
LDL cholesterol, mg/dl	117.4 (26.7)	117.3 (31.7)	0.79	108.8 (27.6)	107.3 (29.6)	0.28
Triglycerides, mg/dl	78.8 (37.6)	98.1 (74.2)	0.007	76.1 (36.2)	82.5 (49.1)	0.04
**Kidney Disease**						
Sustained AER ≥30 mg/24 h, %	7.1	5.3	0.56	3.9	4.9	0.39
**Glycemic Control**						
HbA1c, %	9.2 (1.7)	9.0 (1.6)	0.60	9.0 (1.7)	8.7 (1.5)	<0.001
HbA1c, mmol/mol	76.7 (18.0)	75.1 (17.1)	0.60	75.4 (18.0)	71.2 (16.4)	<0.001

To covert cholesterol to mmol/L, multiply values by 0.0259.
AER=albumin excretion rate, BMI=body mass index, HDL=high-density lipoprotein, LDL=low-density lipoprotein.
[a] P-value for differences by sex by the Wilcoxon rank-sum test for continuous variables or chi-square test for categorical variables.

© 2022 Braffett BH et al. *JAMA Network Open.*

eTable 2. Rate of Change of Cardiometabolic Risk Factors and HbA1c During DCCT/EDIC for Women and Men

	Women (N=680)	Men (N=761)	Women vs. Men Comparison[a]		
	Mean Slope during EDIC Beta (SE)		Difference in Slopes Beta (SE)	t-value	p-value
**Physical**					
BMI, kg/m^2	0.105 (0.007)	0.108 (0.006)	-0.003 (0.009)	-0.36	0.72
Waist circumference, cm	0.266 (0.018)	0.294 (0.017)	-0.028 (0.025)	-1.15	0.25
**Blood Pressure**					
Systolic, mmHg	0.233 (0.015)	0.172 (0.014)	0.061 (0.020)	3.01	0.003
Diastolic, mmHg	0.025 (0.009)	0.045 (0.009)	-0.020 (0.013)	-1.51	0.13
Pulse pressure, mmHg	0.208 (0.011)	0.127 (0.010)	0.081 (0.015)	5.45	<0.001
Pulse rate, bpm	-0.056 (0.013)	0.015 (0.013)	-0.071 (0.018)	-3.92	<0.001
**Lipids**					
Total cholesterol, mg/dl	0.129 (0.051)	0.108 (0.049)	0.021 (0.071)	0.30	0.76
HDL cholesterol, mg/dl	0.241 (0.022)	0.139 (0.021)	0.103 (0.031)	3.31	0.001
LDL cholesterol, mg/dl	-0.145 (0.045)	-0.112 (0.043)	-0.033 (0.062)	-0.53	0.60
Triglycerides, mg/dl[b]	0.003 (0.001)	0.005 (0.001)	-0.003 (0.001)	-2.36	0.02
**Glycemic Control**					
HbA1c, %	-0.006 (0.002)	-0.005 (0.002)	-0.001 (0.003)	-0.24	0.81

ACE=angiotensin-converting enzyme, ARB=angiotensin II receptor blocker, BMI=body mass index, HDL=high-density lipoprotein, LDL=low-density lipoprotein.

[a] Separate LMM models assessing the differences between sexes (women vs. men) in the mean slope of each quantitative risk factor over repeated time points. DCCT/EDIC study year was included in each model as a quantitative random effect. An interaction between sex and study year was included to test the hypothesis of equality of mean slopes. Separate nested models were used to estimate the mean slopes within each sex. Each model was adjusted for DCCT baseline age and treatment group. The mean slope over the duration of the study, difference in slopes (women – men), t-values, and p-values are presented from each model. The signed t-value corresponds to the magnitude and directionality of the association.

[b] Triglyceride values were log transformed.

© 2022 Braffett BH et al. *JAMA Network Open.*

eTable 3. Risk of Cardiovascular Disease in Women vs Men After Individual Adjustment for Cardiometabolic Risk Factors

<u>Time-dependent Covariates</u>	**Any CVD**		**MACE**	
	**Covariate**	**Women vs. Men Adjusted for Covariate**	**Covariate**	**Women vs. Men Adjusted for Covariate**
	Hazard Ratio (95% CI)[a]		Hazard Ratio (95% CI)[a]	
-----	-----	0.87 (0.68, 1.12)	-----	0.79 (0.56, 1.10)
**Physical**				
BMI, per 5 kg/m^2	1.24 (1.06,1.46)	0.90 (0.70,1.15)	1.04 (0.83,1.30)	0.79 (0.56,1.11)
Waist circumference, per 10 cm	1.23 (1.10,1.38)	1.15 (0.86,1.53)	1.07 (0.90,1.26)	0.90 (0.60,1.34)
**Blood Pressure**				
Systolic, per 5 mmHg	1.27 (1.18,1.36)	1.09 (0.85,1.41)	1.25 (1.13,1.38)	0.96 (0.67,1.36)
Diastolic, per 5 mmHg	1.27 (1.13,1.43)	1.04 (0.80,1.35)	1.14 (0.97,1.35)	0.87 (0.61,1.25)
Pulse pressure, per 5 mmHg	1.30 (1.18,1.43)	0.92 (0.72,1.18)	1.33 (1.18,1.51)	0.82 (0.59,1.16)
Pulse rate, per 5 bpm	1.34 (1.22,1.46)	0.75 (0.59,0.97)	1.43 (1.26,1.61)	0.66 (0.47,0.94)
**Lipids**				
Total, per 10 mg/dl	1.12 (1.07,1.18)	0.82 (0.64,1.06)	1.12 (1.05,1.20)	0.74 (0.53,1.04)
HDL, per 10 mg/dl	0.82 (0.73,0.92)	1.09 (0.83,1.42)	0.80 (0.68,0.94)	1.01 (0.69,1.47)
LDL, per 10 mg/dl	1.15 (1.09,1.21)	0.92 (0.72,1.17)	1.14 (1.06,1.23)	0.82 (0.58,1.15)
Triglycerides, per 10%	1.09 (1.06,1.12)	1.00 (0.78,1.28)	1.10 (1.07,1.14)	0.91 (0.65,1.29)
**Medications**				
ACE inhibitor or ARB, yes vs. no	1.07 (0.81,1.40)	0.91 (0.70,1.17)	0.88 (0.61,1.28)	0.82 (0.57,1.16)
β-blockers, yes vs. no	2.02 (1.36,3.00)	0.90 (0.69,1.15)	2.50 (1.59,3.93)	0.81 (0.57,1.15)
Calcium channel blockers, y vs. n)	1.65 (1.13,2.41)	0.91 (0.70,1.17)	2.00 (1.25,3.19)	0.83 (0.59,1.18)
Lipid-lowering, yes vs. no	0.98 (0.73,1.30)	0.90 (0.70,1.16)	0.77 (0.52,1.13)	0.80 (0.56,1.14)
**Glycemic Control**				
HbA1c, per 1%	1.57 (1.40,1.76)	0.82 (0.64,1.05)	1.80 (1.55,2.10)	0.71 (0.50,1.00)

ACE=angiotensin-converting enzyme, ARB=angiotensin II receptor blocker, BMI=body mass index, HDL=high-density lipoprotein, LDL=low-density lipoprotein.

[a] Separate Cox proportional hazard regression models assessing the difference between sexes (women vs. men) on the risk of cardiovascular disease, after separate adjustments for each cardiometabolic risk factor as a time-dependent covariate. Each model was further adjusted for DCCT baseline age and treatment group. The hazard ratios (95% CI) for the time-dependent covariate and for the sex effect are presented from each model.

© 2022 Braffett BH et al. *JAMA Network Open.*

eTable 4. Associations Between Risk Factor Levels or Targets and Cardiovascular Disease Risk During DCCT/EDIC in Women and Men

			Any CVD		MACE	
	**Women**	**Men**	**Women**	**Men**	**Women**	**Men**
	Mean (SD) or %		Hazard Ratio (95% CI)[a]		Hazard Ratio (95% CI)[a]	
**Risk Factor Targets**						
HDL ≥50 mg/dl women, ≥40 mg/dl men	74.3	86.6	0.76 (0.49,1.17)	0.60 (0.40,0.88)	0.54 (0.30,0.96)	0.50 (0.30,0.83)

[a] Separate stratified Cox proportional hazard regression models adjusted for DCCT baseline age and treatment group. The hazard ratios (95% CI) for the time-dependent covariate are presented from each model. An interaction between sex and risk factor was evaluated in each model.

© 2022 Braffett BH et al. *JAMA Network Open.*

eFigure. Mean Slope During EDIC for Women vs Men

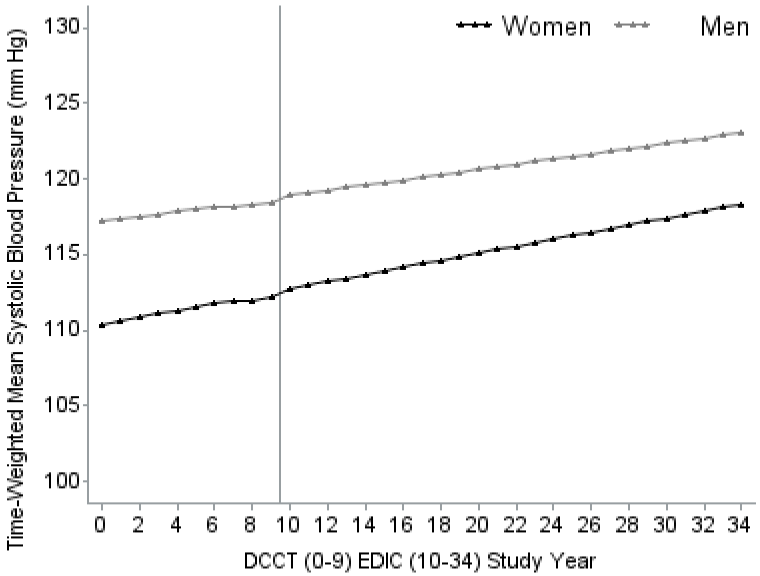

A.

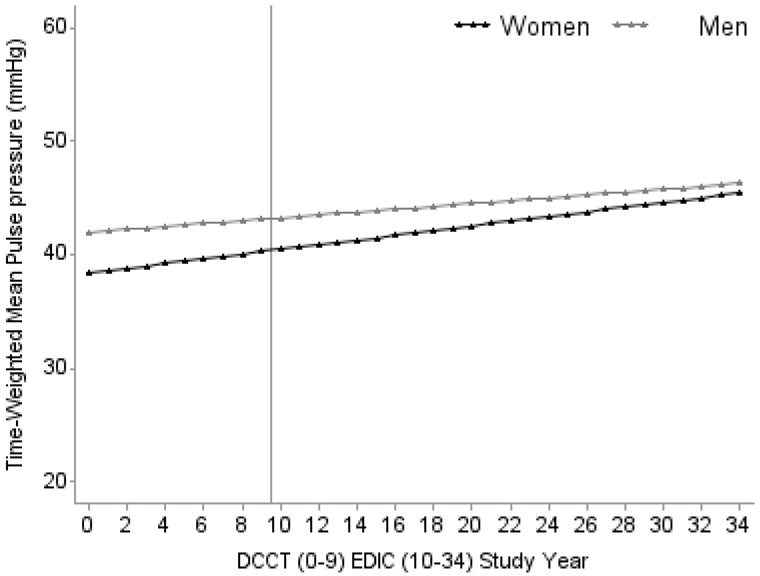

B.

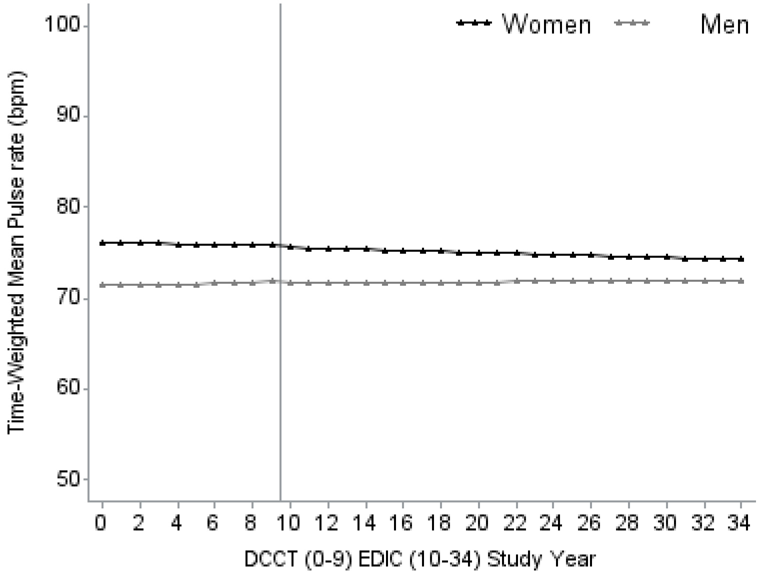

C.

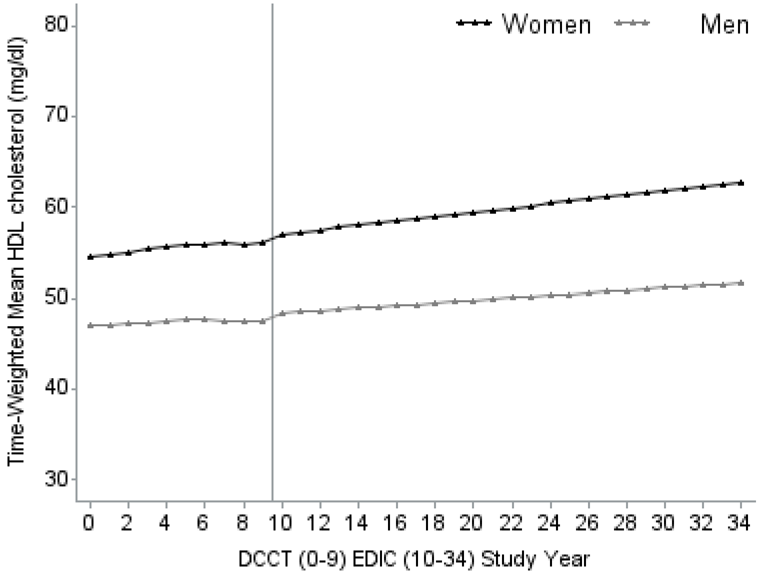

D.

Data are from LMM models assessing the differences between women and men in the mean slope of each quantitative risk factor over repeated time points. Each model was adjusted for DCCT baseline age and treatment group.

© 2022 Braffett BH et al. *JAMA Network Open.*

eAppendix. DCCT/EDIC Research Group

Study Chairpersons – D.M. Nathan (chair), B. Zinman (vice-chair); *Past*: O. Crofford; *Deceased*: S. Genuth

Editor, EDIC Publications – D.M. Nathan

Clinical Centers

Case Western Reserve University – *Current*: R. Gubitosi-Klug, L. Mayer, J. Wood, D. Miller, A. Nayate, M. Novak, S. Pendegast, L. Singerman, D. Weiss, H. Zegarra; *Past*: E. Brown, P. Crawford, M. Palmert, P. Pugsley, J. Quin, S. Smith-Brewer; *Deceased*: W. Dahms, S. Genuth, J. McConnell

Weill Cornell Medical College – *Current*: N.S. Gregory, R. Hanna, R. Chan, S. Kiss, A. Orlin, M. Rubin; *Past*: S. Barron, B. Bosco, D. Brillon, S. Chang, A. Dwoskin, M. Heinemann, L. Jovanovic, M.E. Lackaye, T. Lee, B. Levy, V. Reppucci, M. Richardson; *Deceased*: R. Campbell

Henry Ford Health System – *Current*: A. Bhan, J.K. Jones, D. Kruger, P.A. Edwards, H. Remtema; *Past*: E. Angus, A. Galprin, M. McLellan, A. Thomas; *Deceased*: J.D. Carey, F. Whitehouse

International Diabetes Center – *Current*: R. Bergenstal, S. Dunnigan, M. Johnson, A. Carlson, ; *Past*: R. Birk, P. Callahan, G. Castle, R. Cuddihy, M. Franz, D. Freking, L. Gill, J. Gott, K. Gunyou, P. Hollander, D. Kendall, J. Laechelt, S. List, G. Matfin, W. Mestrezat, J. Nelson, B. Olson, N. Rude, M. Spencer, L. Thomas; *Deceased*: D. Etzwiler, K. Morgan

Joslin Diabetes Center – *Current*: L.P. Aiello, E. Golden, P. Arrigg, R. Beaser, L. Bestourous, J. Cavallerano, R. Cavicchi, O. Ganda, O. Hamdy, T. Murtha, D. Schlossman, S. Shah, G. Sharuk, P. Silva, P. Silver, M. Stockman, J. Sun, E. Weimann; *Past*: V. Asuquo, A. Jacobson, R. Kirby, L. Rand, J. Rosenzwieg, H. Wolpert

Massachusetts General Hospital – *Current*: D.M. Nathan, M.E. Larkin, M. Cayford, A. deManbey, L . Gurry, J. Heier, A. Joseph, F. Leandre, K. Martin, C. Shah, C. Stevens, N. Thangthaeng; *Past*: E. Anderson, H. Bode, S. Brink, M. Christofi, C. Cornish, D. Cros, S. Crowell, L. Delahanty, K. Folino, S. Fritz, C. Gauthier-Kelly, J. Godine, C. Haggan, K. Hansen, P. Lou, J. Lynch, C. McKitrick, D. Moore, D. Norman, M. Ong, E. Ryan, C. Taylor, D. Zimbler

Mayo Clinic – *Current*: A. Vella, A. Zipse, A. Barkmeier; *Past*: B. French, M. Haymond, J. Mortenson, J. Pach, R. Rizza, L. Schmidt, W.F. Schwenk, F.J. Service, R. Woodwick, G. Ziegler; *Deceased*: R. Colligan, A. Lucas, B. Zimmerman

Medical University of South Carolina – *Current*: H. Karanchi, L. Spillers, J. Fernandes, K. Hermayer, S. Kwon, K. Lee, M. Lopes-Virella, T. Lyons, M. Nutaitis; *Past*: A. Blevins, M. Bracey, S. Caulder, J. Colwell, S. Elsing, A. Farr, D. Lee, P. Lindsey, L. Luttrell, R. Mayfield, J. Parker, N. Patel, C. Pittman, J. Selby, J. Soule, M. Szpiech, T. Thompson, D. Wood, S. Yacoub-Wasef

Northwestern University – *Current*: A. Wallia, M. Hartmuller, S. Ajroud-Driss, P. Astelford, A. Degillio, M. Gill, L. Jampol, C. Johnson, L. Kaminski, N. Leloudes, A. Lyon, R. Mirza, D. Ryan, E. Simjanoski, Z. Strugula; *Past*: D. Adelman, S. Colson, M. Molitch, B. Schaefer

University of California, San Diego – *Current*: S. Mudaliar, G. Lorenzi, O. Kolterman, M. Goldbaum; *Past*: T. Clark, M. Giotta, I. Grant, K. Jones, R. Lyon, M. Prince, R. Reed, M. Swenson; *Deceased*: G. Friedenberg

University of Iowa – *Current*: W.I. Sivitz, B. Vittetoe, J. Kramer; *Past*: M. Bayless, C. Fountain, R. Hoffman, J. MacIndoe, N. Olson, H. Schrott, L. Snetselaar, T. Weingeist, R. Zeitler

University of Maryland – *Current*: R. Miller, S. Johnsonbaugh; *Past*: M. Carney, D. Counts, T. Donner, J. Gordon, M. Hebdon, R. Hemady, B. Jones, A. Kowarski, R. Liss, S. Mendley, D. Ostrowski, M. Patronas, P. Salemi, S. Steidl

University of Michigan – *Current*: W.H. Herman, R. Pop-Busui, C.L. Martin, P. Lee, J. W. Albers, E.L. Feldman; *Past*: N. Burkhart, D.A. Greene, T. Sandford, M.J. Stevens; *Deceased*: J. Floyd

University of Minnesota – *Current*: J. Bantle, M. Rhodes, D. Koozekanani, S. Montezuma, J. Terry; *Past*: N. Flaherty, F. Goetz, C. Kwong, L. McKenzie, M. Mech, J. Olson, B. Rogness, T. Strand, R. Warhol, N. Wimmergren

University of Missouri – *Current*: D. Goldstein, D. Hainsworth, S. Hitt; *Deceased*: J. Giangiacomo

University of New Mexico – *Current*: D.S. Schade, J.L. Canady, R.B. Avery, M.R. Burge, J.E. Chapin, A. Das, L.H. Ketai; *Past*: D. Hornbeck, C. Johannes, J. Rich, M.L Schluter

University of Pennsylvania – *Current*: M. Schutta, P.A. Bourne, A. Brucker; *Past*: S. Braunstein, B.J. Maschak-Carey, S. Schwartz; *Deceased*: L. Baker

University of Pittsburgh – *Current*: T. Orchard, B.A. Coons, D. Rubinstein; *Past*: D. Becker, L. Cimino, B. Doft, D. Finegold, K. Kelly, L. Lobes, N. Silvers, T. Songer, D. Steinberg, L. Steranchak, J.Wesche; *Deceased*: A. Drash

University of South Florida – *Current*: J.I. Malone, A. Morrison, M.L. Bernal, P.R. Pavan; *Past*: L. Babbione, T.J. DeClue, N. Grove, D. McMillan, H. Solc, E.A. Tanaka, J. Vaccaro-Kish

University of Tennessee – *Current*: S. Dagogo-Jack, C. Wigley, S. Huddleston, A. Patel; *Past*: M. Bryer-Ash, E. Chaum, A. Iannacone, H. Lambeth, D. Meyer, S. Moser, M.B. Murphy, H. Ricks, S. Schussler, S. Yoser; *Deceased*: A. Kitabchi

University of Texas – *Current*: P. Raskin, S. Strowig, YG. He, E. Mendelson, RL. Ufret-Vincenty; *Past*: M. Basco; *Deceased*: S. Cercone

University of Toronto – *Current*: B.A. Perkins, B. Zinman, A. Barnie, N. Bakshi, M. Brent, R. Devenyi, K. Koushan, M. Mandelcorn, F. Perdikaris, L. Tuason; *Past*: D. Daneman, R. Ehrlich, S. Ferguson, A. Gordon, K. Perlman, S. Rogers

University of Washington – *Current*: I. Hirsch, R. Fahlstrom, L. Van Ottingham, I.H. de Boer, L. Olmos de Koo; *Past*: S. Catton, J. Ginsberg, J. Kinyoun, J. Palmer

University of Western Ontario – *Current*: C. McDonald, M. Driscoll, J. Bylsma, T. Sheidow; *Past*: W. Brown, C. Canny, P. Colby, S. Debrabandere, J. Dupre, J. Harth, I. Hramiak, M. Jenner, J. Mahon, D. Nicolle, N.W. Rodger, T. Smith

Vanderbilt University – *Current*: M. May, J. Lipps Hagan, T. Adkins, A. Agarwal, C. Lovell; *Past*: S. Feman, R. Lorenz, R. Ramker; *Deceased*: L. Survant

Washington University, St. Louis – *Current*: N.H. White, L. Levandoski; *Deceased*: I. Boniuk, J. Santiago

Yale University – *Current*: W. Tamborlane, P. Gatcomb, K. Stoessel; *Past*: J. Ahern, K. Fong, P. Ossorio, P. Ramos

Albert Einstein – *Past*: J. Brown-Friday, J. Crandall, H. Engel, S. Engel, H. Martinez, M. Phillips, M. Reid, H. Shamoon, J. Sheindlin

Clinical Coordinating Center

Case Western Reserve University – *Current*: R. Gubitosi-Klug, L. Mayer, C. Beck, K. Farrell, P. Gaston; *Past*: S. Genuth, M. Palmert, J. Quin, R. Trail; *Deceased*: W. Dahms

Data Coordinating Center

George Washington University, The Biostatistics Center – *Current*: J. Lachin, I. Bebu, B. Braffett, J. Backlund, L. Diminick, L. El ghormli, X. Gao, S. Ho, D. Kenny, K. Klumpp, M. Lin, V. Trapani; *Past*: K. Anderson, K. Chan, P. Cleary, A. Determan, L. Dews, W. Hsu, P. McGee, H. Pan,B. Petty, D. Rosenberg, B. Rutledge, W. Sun, S. Villavicencio, N. Younes; *Deceased*: C. Williams

National Institute of Diabetes and Digestive and Kidney Disease

National Institute of Diabetes and Digestive and Kidney Disease Program Office – *Current*: E. Leschek; *Past*: C. Cowie, C. Siebert

EDIC Core Central Units

Central Biochemistry Laboratory (University of Minnesota) – *Current*: M. Steffes, A. Karger, J. Seegmiller, V. Arends; *Past*: J. Bucksa, B. Chavers, A. Killeen, M. Nowicki, A. Saenger

Central ECG Reading Unit (Wake Forest School of Medicine) – *Current*: E.Z. Soliman, M. Barr, C. Campbell, S. Hensley, J. Hu, L. Keasler, Y. Li, T. Taylor, Z.M. Zhang; *Past*: Y. Pokharel, R. Prineas

Central Ophthalmologic Reading Unit (University of Wisconsin) – *Current*: B. Blodi, R. Danis, D. Lawrence, H. Wabers; *Past*: M. Burger, M. Davis, J. Dingledine, V. Gama, S. Gangaputra, L. Hubbard, S. Neill, R. Sussman

Central Neuropsychological Reading Unit (NYU Long Island School of Medicine, University of Pittsburgh) – *Current*: A. Jacobson, C. Ryan, D. Saporito; *Past*: B. Burzuk, E. Cupelli, M. Geckle, D. Sandstrom, F. Thoma, T. Williams, T. Woodfill

© 2022 Braffett BH et al. *JAMA Network Open.*
